# Dietary triglycerides profoundly affect oral sensitization to peanut protein in an adjuvant-free mouse model of peanut allergy

**DOI:** 10.1186/2045-7022-1-S1-O33

**Published:** 2011-08-12

**Authors:** Erik Eckhardt, Jianing Li

**Affiliations:** 1 University of Kentucky, Internal Medicine, Lexington, USA; 2 University of Kentucky, Graduate Center for Nutritional Sciences, Lexington, USA

## Background and aim

Peanuts and peanut butter contain significant amounts of long-chain triglycerides (LCT), which stimulate lymphatic chylomicron transport. We have recently reported that intestinal absorption of a dietary antigen (ovalbumin; (OVA)) is significantly enhanced when chylomicron formation is induced. This prompted us to investigate the effect of LCT on oral sensitization to concomitantly ingested protein antigens.

## Methods

To induce peanut allergy, C3H/HEJ mice were gavaged with one dose of peanut butter of which the fat was replaced with MCT (medium-chain triglycerides), LCT (peanut or soy oil), or LCT plus the chylomicron inhibitor Pluronic L-81. Anti-peanut IgE was measured 18 days later, and mice were then challenged intraperitoneally with peanut extract. Body temperature was measured every five minutes, and plasma was collected after 90 minutes to detect mmcp-1 (a marker for histamine release). In separate experiments with BALB/c mice, we tested the effect of dietary triglycerides on oral tolerance to OVA. Oral tolerance was reflected by aAnti-OVA IgG levels after systemic sensitization following OVA feeding in the context of altered triglyceride composition.

## Results

Gavage of peanut butter proteins in MCT induced significant anti peanut-protein IgE responses and anaphylaxis upon peanut challenge, as reflected by a rapid drop in temperature and substantial plasma mmcp-1 release. Gavage with LCT almost completely protected from allergy and anaphylaxis, but this was completely reversed by addition of Pluronic L-81 during gavage, suggesting that chylomicron formation during oral sensitization was protective. Similarly, oral tolerance to OVA was abrogated when OVA was fed with MCT or with Pluronic L-81.

## Conclusion

Postprandial chylomicron secretion significantly affects the immune response to concomitantly ingested dietary allergens. We speculate that postprandial chylomicronemia promotes mesenteric lymph node transport of dietary allergens, which may dampen allergenicity by promoting oral tolerance.

